# Cognitive Intervention Using Information and Communication Technology for Older Adults with Mild Cognitive Impairment: A Systematic Review and Meta-Analysis

**DOI:** 10.3390/ijerph182111535

**Published:** 2021-11-02

**Authors:** Ae-Ri Jung, Dasom Kim, Eun-A Park

**Affiliations:** 1 College of Nursing, Bucheon University, Bucheon 14774, Korea; aeri83@bc.ac.kr (A.-R.J.); pea0701@bc.ac.kr (E.-A.P.); 2 Nowon-gu Public Health Center, 437, Nohae-ro, Nowon-gu, Seoul 01689, Korea

**Keywords:** systematic review, meta-analysis, cognitive dysfunction, dementia, cognitive aging, memory disorders, aged, technology, mobile health, virtual reality

## Abstract

Background: Outside activities have decreased due to the spread of the COVID-19 since 2019; therefore, the need for education using information and communication technology (ICT) for older adults with mild cognitive impairment (MCI) has increased. This study systematically evaluated the effects of cognitive enhancement interventions using ICT on older adults with MCI. Methods: Six electronic databases (CINAHL, Cochrane CENTRAL, EMBASE, PubMed, RISS, and KISS) were searched for relevant articles published from 25 January to 10 February, 2021. Results: As a result of the systematic literature review, 12 research papers were finally selected as the literature for quality evaluation, and 11 final papers were selected, excluding one in the quality evaluation. From the synthesis in this study, it was found that cognitive intervention using ICT showed a statistically significant positive effect on cognitive function when compared with various control groups (SMD = 0.4547; *p* < 0.001; 95% CI: 0.1980–0.7113). Conclusions: Through this study, cognitive intervention using ICT showed a small effect size for older adults with mild cognitive impairment, and statistically significant results were found.

## 1. Introduction

Mild cognitive impairment (MCI) is a stage between normal cognitive function and dementia, where an older adults’ intellectual and daily life performance abilities are maintained, but have significantly reduced memory and partial cognitive impairments in language ability, space–time classification, and frontal function [1]. Individuals with MCI are included in the high-risk group for dementia, because they are 10 to 15 times more likely to develop dementia than those with normal cognitive function [2]. In addition, subjective memory complaints (SMCs) in older adults are a significant independent predictor for a dementia diagnosis [3].

MCI can be divided into amnestic and non-amnestic forms [4]. In the former case, memory is primarily impaired, and the individual forgets important information that would normally be easily remembered. In the latter case, executive functions are impaired, such as the ability to make sound decisions, judge the time or sequence of steps needed to complete a complex task, or visual perception [5].

MCI is a starting point for clinically diagnosing the progression of cognitive decline; therefore, it is important to reduce morbidity and the burden on caregivers by delaying the progression to dementia through early diagnosis and intervention [6]. Older adults with MCI prefer non-drug interventions and are aware of their importance because it is difficult to find drugs that have proven long-term therapeutic effects [7,8]. Moreover, in previous studies, it has been reported that non-drug interventions are effective in improving cognitive function in older adults with MCI [9]. Furthermore, combined cognitive-physical intervention showed more positive effect on cognitive reinforcement than a single-domain intervention [9,10]. For cognitive-enhanced interventions, most studies have shown a direct relation with the intervention period, that is, longer intervention periods being more effective. However, the standardized intervention cycle, frequency of each intervention program and the persistence of post-intervention effects differ between studies, and the basis for this is not clear [11].

According to a previous study analyzing past interventions [12], multi-domain cognitive training programs are more effective in increasing neuroplasticity. In most studies, a single mode of therapy, such as occupational therapy, kinesiotherapy, or art therapy, was applied [2], and cognitive training only targeted a single cognitive domain, such as memory [13,14]. A single-domain cognitive intervention might have theoretical importance because it may allow researchers to investigate direct training-related effects [15], but multi-domain training could potentially have more practical advantages because multiple cognitive functions are required for humans to survive [16]. It is therefore possible that multi-domain cognitive training could elicit more synergistic transfer effects across domains than single-domain cognitive training [17,18].

In recent years, there has been rapid development in information and communication technology (ICT), and the COVID-19 pandemic is expected to further increase the demand for cognitive enhancement programs using ICT. Thus, systematic literature and meta-analysis studies are timely and will serve as a basis for judging the applicability in various sites. Nevertheless, there have been no systematic literature reviews that examine the effect of ICT-based cognitive function enhancement programs on cognitive functions in older adults with MCI, and no comprehensive integration of their effects has been made.

Therefore, to verify the effectiveness of cognitive training programs using ICT for older adults with MCI, this study aims to examine previous studies through a systematic literature review, synthesize and analyze the effect size of the intervention using meta-analysis, and suggest paradigms for future studies.

The objective of this systematic review was to synthesize and analyze the indicators of effectiveness and results of ICT interventions in older adults of the community with MCI.

## 2. Materials and Methods

### 2.1. Design

This study was a systematic review and meta-analysis that integrated and analyzed the contents and results of cognitive intervention studies using ICT for older adults with MCI.

### 2.2. Search Strategy

This systematic review registered in PROSPERO (ID: CRD42021279093) was conducted following the reporting items for systematic reviews and meta-analyses specified by the PRISMA checklist. The research question here was ‘Is nursing intervention using ICT effective for cognitive function of older adults with MCI?’ The participants (P) in this study were older adults, intervention (I) was the nursing intervention using ICT, and the outcome (O) was cognitive function.

The research question was prepared in the form of PICOTS-SD (Participants, Interventions, Comparisons, Outcomes, Timing of outcome measurement, Setting, Study Design) (Table 1). The selection criteria for the systematic literature review were limited to randomized controlled trials (RCTs), and non-randomized controlled trials (NRCTs) were excluded. The effect size derived from NRCTs may be overestimated or underestimated; therefore, studies suggest that the reliability of their results can be further increased by identifying the effect only through an RCT study when synthesizing the research effect [18,19,20,21].

Literature selection and data extraction were carried out by three researchers, and in cases of disagreement, selection was performed through discussion at research meetings. The criteria for the selection of studies corresponded to the above PICOTS-SD, and there were no exclusion criteria based on the number or duration of interventions. In addition, no restrictions were placed on the outcome variables or duration. Literature reviews were not excluded from the search stage, but only the primary studies confirmed in the literature review were included in the analysis of papers that was used as data for this study.

Inclusion criteria:

① (P) people with a diagnosis of MCI or SMC;
② (P) diagnosis of MCI using the Petersen criteria or revised Petersen criteria [1,22,23,24], Clinical Dementia Rating Scale (CDR) = 0.5 [25,26,27], the Consortium to Establish a Registry for Alzheimer’s Disease Assessment Packet (CERAD) [28], or a combination;
③ (P) when the inclusion standard was unclear and required discussion, we accepted the MCI and SMC status as defined by the authors of each trial. These could include diagnostic assessment and/or subjective memory complaints with reduced scores on cognitive tests such as the Mini Mental State Examination (MMSE) or Montreal Cognitive Assessment (MoCA);
④ (P) recruited and clinically classified as having MCI at time of performing the test were eligible for inclusion;
⑤ (I) ICT interventions were included in a broad manner that could include PCs, desktops, laptops, handheld devices, and other types of wireless or cable-connected equipment [29];
⑥ (O) studies which used cognitive function as a primary outcome.

Exclusion criteria:

① (P) not subject to MCI (mean age > 65 years);
② (P) who were diagnosed with a mood disorder, such as dementia or depression, and on drug treatment;
③ (P) diagnosed with stroke, Parkinson’s disease, schizophrenia, epilepsy, and other neuropsychiatric disorders;
④ (I) did not use ICT as a major intervention;
⑤ (O) studies in which cognitive function, the primary outcome variable, could not be confirmed;
⑥ Full text was unavailable;
⑦ Not related to humans, protocols without research results, editorial comments, among others;
⑧ Written in a language other than English or Korean;
⑨ Published before 2010.

### 2.3. Data Collection

Research papers applying cognitive training using ICT for older adults with MCI were collected by searching several databases from 25 January 2021 to 10 February 2021. MEDLINE (PubMed), EMBASE, and Cochrane CENTRAL, which correspond to the core databases of the COSI model, were searched. CINAHL, a specialized database for nursing and health medical literature, was also included. Korean databases KISS and RISS were also included. The search terms used differed across the selected databases; therefore, the structure of MeSH search terms for MEDLINE and Cochrane CENTRAL, EMTREE for EMBASE, and CINAHL headings structure for CINAHL were identified. After reviewing 100 abstracts obtained using MeSH keywords in MEDLINE, alternative words were added to create a concept map of the search terms (Table 2) and improve the sensitivity of search terms. A search query for each database was constructed using a combination of [Participants] AND [Intervention] AND [Study design] search terms, and a region filter was added to include search terms in the title and abstract to improve the search specificity. Databases that have Boolean operators and Truncation & Wildcard were used, and the search results were limited to human participants and literature published from 2010 to 2021 (see Supplementary Materials, Table S1).

In the process of reviewing the full texts, if there were any related documents that were not included in the search results, the references were manually searched and confirmed. The study selection process was independently conducted by three reviewers who had experience in publishing systematic literature review papers.

### 2.4. Risk-of-Bias Assessment

Study bias was evaluated using the Cochrane Risk of Bias (RoB) tool 2.0 [30], a tool for evaluating bias in randomized trials. The quality of the studies was evaluated independently by three researchers, and in cases of disagreement, mutual agreement was reached through a research meeting. In the case of RoB, all studies included in the full-text review were evaluated, and six items were used as the major category. The six major categories were random sequence generation, allocation concealment, blinding of outcome assessment, incomplete outcome data (attrition bias), and selective reporting.

### 2.5. Data Synthesis

The focus of data synthesis is to integrate the effect index and outcome of cognitive function and depression for ICT-based cognitive training in older adults with MCI and to present an appropriate evaluation index. Therefore, data extraction was performed using a structured table for the target studies that met the inclusion criteria. Data extraction included publication year, country, setting, design, population, inclusion criteria, exclusion criteria, training characteristics, outcome measures, and results.

Statistical analysis was performed using the Cochrane Review Manager software 5.3.2 (RevMan) [31]. Studies used various ICT training devices, such as iPads and virtual reality (VR); therefore, the average effect and 95% confidence intervals (CIs) of the outcome variables were analyzed using a random effects model. In addition, the study results were synthesized using different cognitive evaluation tools, and the standardized mean difference (SMD) was generated.

In addition, subgroup analysis and cumulative sensitivity analysis were per-formed. Subgroup analysis compared studies using multiple interventions and single interventions, active control group and passive control group, respectively. Research classified as an active control group includes cases in which cognitive interventions are provided or information on cognitive programs developed by researchers is provided. Studies classified as a passive control group included cases in which no intervention was provided, or discussions, simple material, and other educational programs were provided.

According to Cohen, the effect size is classified as a large effect size when the SMD is 0.8 or more, a medium effect size when it is 0.5–0.8, and a small effect size when it is 0.2–0.5 [32]. The Higgins I² test and Cochrane Q statistics were assessed to determine the heterogeneity between studies [33]. I-squared test results below 25%, 50–75%, and above 75% are considered to signify low, moderate, and high statistical heterogeneity, respectively. Cochrane Q statistics that indicate *p*-values below 0.10 are considered to be significantly positive in terms of statistical heterogeneity. Although funnel plots are not suitable for confirming publication bias in fewer than 10 studies, they were plotted to understand visual trends in reporting bias [21].

## 3. Results

### 3.1. Study Characteristics

**Figure 1:**
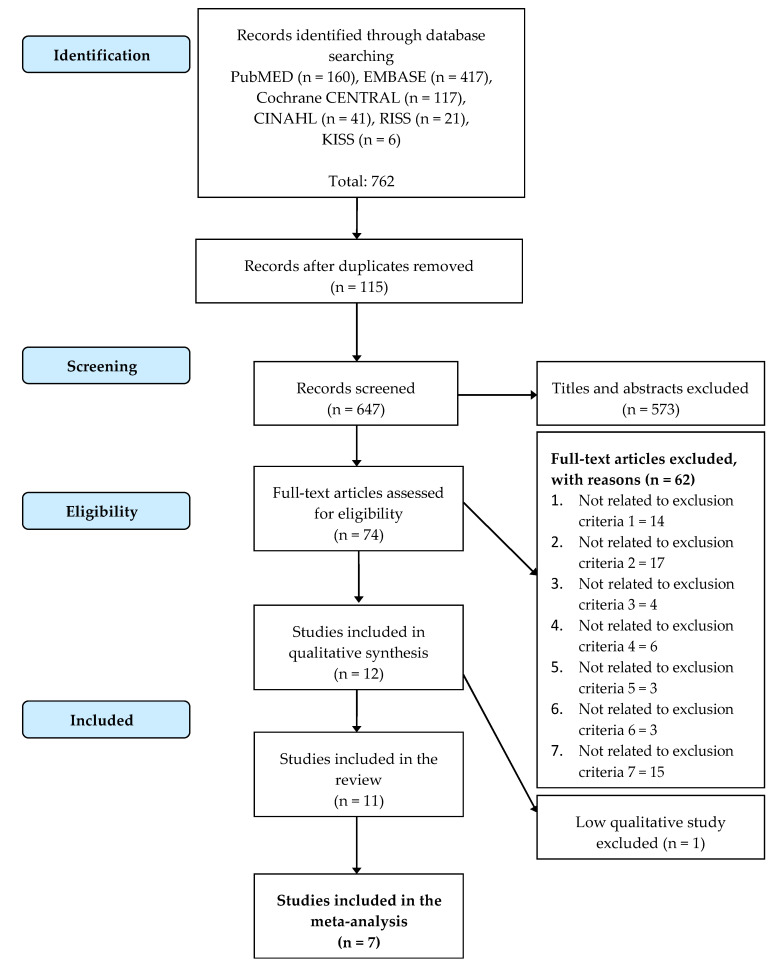
PRISMA flow chart of study selection.

A total of 762 studies were retrieved using different databases: CINAHL (41), Cochrane CENTRAL (117), EMBASE (417), PubMed (160), RISS (12), and KISS (6). We excluded 115 duplicated studies and then screened the titles and abstracts of the remaining 647 non-duplicated studies, after which we excluded 573 studies. Following the full-text review of 74 studies, 12 studies were included in this review for quality evaluation. As a result of the quality evaluation, 11 final studies were selected, and one study [34] was excluded (Figure 1) [35].

The characteristics of the included studies are presented in Table 3. Of the eleven studies, seven were conducted between 2016 and 2019, and four were conducted after 2020. Three studies were conducted in Korea, two each in France and the United States, and one each in China, Greece, Taiwan, and Thailand.

In terms of the research setting, four studies were conducted at a medical center, and seven studies were conducted in the community. Eight studies were designed as two-arm studies, two were three-arm studies, and one was a five-arm parallel group trial. Regarding the number of participants, four studies included fewer than 50 people, three studies had between 50 and 100 people, four studies had more than 100 people, with a total of 829 participants included in this meta-analysis. The age range of the study participants was 65.7 to 80.9 years.

### 3.2. Quality Evaluation of Selected Studies

**Figure 2:**
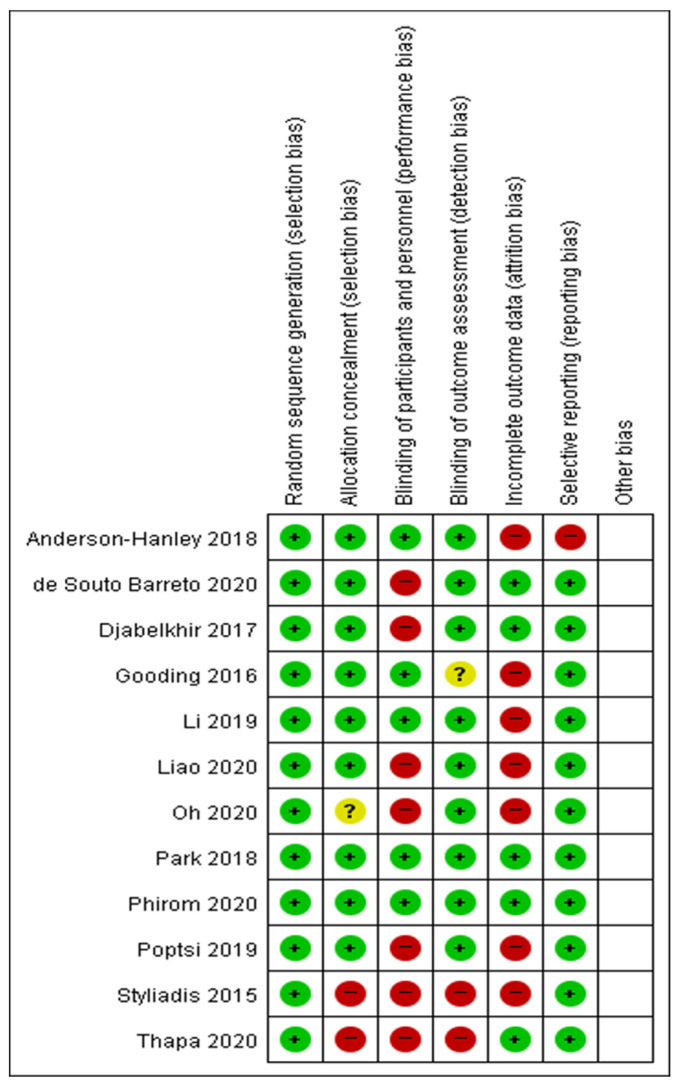
Risk of bias assessments. Green: Low risk, Yellow: Unclear, Red: High risk.

Figure 1 shows the results of the risk of bias quality evaluation for selected studies. For each evaluation domain, three levels were evaluated and labeled as the following: ‘high’, if there was an obvious risk; ‘low’, if there was no obvious risk; and ‘unclear’, if the risk was uncertain or not described in the paper. In papers in which four or more items were found to be ‘high risk’, through author agreement, we decided to exclude them as papers for systematic review and data ex-traction. Accordingly, a total of 11 papers [36,37,38,39,40,41,42,43,44,45,46] were included except Styliadis (2015) for systematic review and data extraction (Figure 2).

### 3.3. Reporting Bias

**Figure 3:**
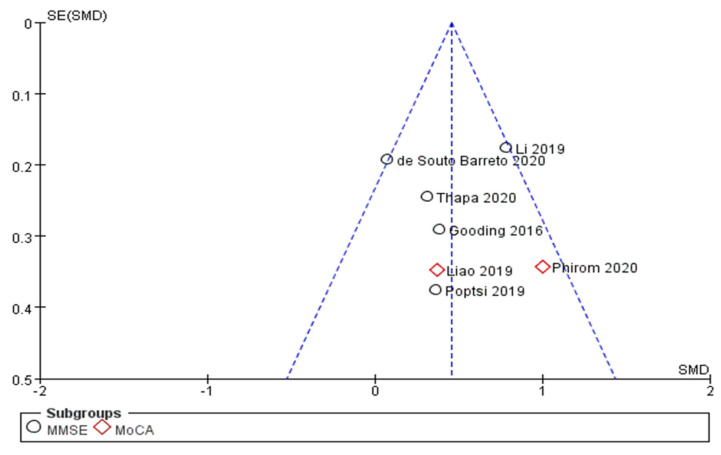
Funnel plot of selected studies.

No clear asymmetry can be observed in the funnel plot (Figure 3). However, because fewer than 10 studies were analyzed, it may show symmetry by chance [21]. Additionally, Egger’s test and Begg’s test showed no statistically significant publication bias (Egger’s test t = 0.74, *p* = 0.475; Begg’s test z = 0.00, *p* = 1.00). However, it is not recommended to conclude publication bias with quantitative results when fewer than 10 studies are included. Thus, additional RCTs on this topic are needed to clearly identify publication bias.

### 3.4. Characteristics of the Training Sessions

The studies were conducted using seven individual programs, three group programs, and one individual and group mixed program. Moreover, seven studies were multi-domain studies that stimulated physical and cognitive function (Table 4).

The training devices were as follows: four studies used a computer, two studies each used VR or a tablet, and one study each used a Nintendo Wii, Xbox 360 Kinect Sensor V2, and a videogame.

Regarding the training time per session, two were between 10 and 30 min, two were between 30 and 60 min, and six were more than 60 min. Regarding the training duration, one lasted 4 weeks, one lasted 8 weeks, four lasted less than 12 weeks, one lasted 16 weeks, and four lasted 24 weeks. Regarding the frequency of training sessions, one was once per week, four were twice per week, and six were more than three times per week. Additionally, training instructors such as neuropsychologists, physiotherapists, and psychologists were included.

### 3.5. Measurement Variables and Tools for Selected Studies

As a result of the study, many psycho-psychological variables, such as depression, physical activity, and quality of life, as well as physiological variables, including cognitive function, were measured.

As a tool to measure global cognitive function, MMSE was used in seven studies, and MoCA was used in three studies. For cognitive function, variables of executive function, attention, and visuo-spatial memory were measured using various cognitive function measurement tools. In cases of multi-domain training, physical functions such as strength and speed were also measured to verify the effect. In addition, the effectiveness of the program was measured in various aspects using psychological measurement tools for depression, anxiety, quality of life, and physiological measurement tools such as electroencephalogram (EEG), and magnetic resonance imaging (MRI) (Table 5).

### 3.6. Data Synthesis

Data were extracted from 11 selected papers after quality evaluation; individual studies used various tools such as MMSE, MoCA, Rey Auditory Verbal Learning Test (RAVLT), and Repeatable Battery for the Assessment of Neuropsychological Status (RBANS) to evaluate cognitive impairment. For meta-analysis, the researcher checked the original text of each tool and tried to select and synthesize a tool with less heterogeneity. In the cases of MMSE and MoCA, similar categories such as orientation, memory registration, memory recall, and attention were measured. Papers excluded from the analysis were ‘difficult to clearly extract data between the experimental group and the control group’ [36], ‘papers where both the control group and the intervention group used ICT’ [38,43], or ‘the content of the intervention in the two groups was the same, but only the intervention provider was different’ [42]. Accordingly, after excluding these four studies, seven studies were included in the final meta-analysis.

**Figure 4:**
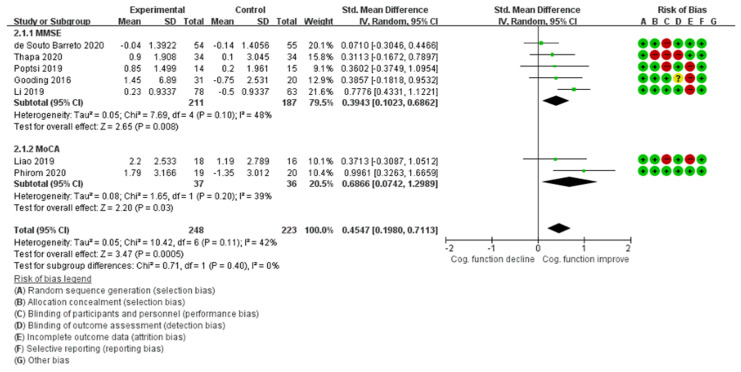
Forest plots of global cognition of all selected studies.

As a result of the synthesis of studies, it was found that the group which received the cognitive intervention using ICT showed a statistically significant positive effect on cognitive function when compared with the various control groups (SMD = 0.4547; *p* < 0.001; 95% CI: 0.1980–0.7113; Figure 4). $I^{2}$ was 42% and the Cochrane Q statistic was not statistically significant ($\chi^{2}=10.42$, *p* = 0.11) [47]. The SMD of all studies was positive. The effect size in Figure 4 indicates the overall effect size of cognitive intervention using ICT when the period, frequency, moderator, and type of intervention performed in the control group were not considered.

**Figure 5:**
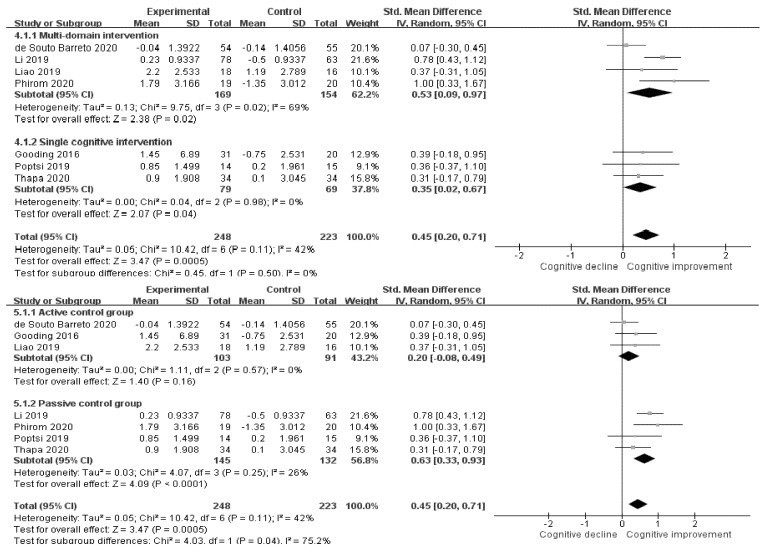
Forest plots of subgroup analysis.

We compared multidomain intervention studies with single cognitive intervention studies through subgroup analysis (Figure 5). As a result, the pooled SMD of the multidomain intervention was 0.53 (CI: 0.09–0.97, *p* = 0.02), and the single cognitive intervention was 0.35 (CI: 0.02–0.67, *p* = 0.04). Additionally, when comparing studies using the active control group (ACG) and the passive control group (PCG), the SMD of the ACG studies was 0.20 (CI: −0.08–0.49, *p* = 0.16), and the SMD of the PCG studies was 0.63 (CI: 0.33–0.94, *p* < 0.001).

**Figure 6:**
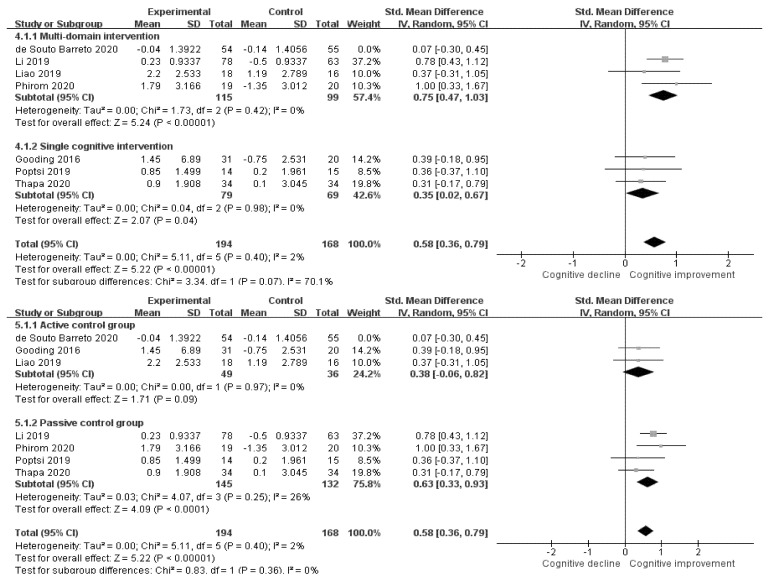
Forest plots of subgroup analysis after excluding heterogeneous study.

We identified studies with high heterogeneity by performing cumulative sensitive analysis. The study of de Souto Barreto [37] was identified with high heterogeneity. Therefore, in Figure 6, a forest plot without de Souto Barreto’s study [37] is presented. As a result, the SMD of multidomain intervention was 0.75 (CI: 0.47–1.03, *p* < 0.001), and the SMD of single cognitive intervention was 0.35 (CI: 0.02–0.67, *p* = 0.04). Both subgroups showed statistically significant effects on cognition. The SMD of the ACG studies was 0.38 (CI: −0.06–0.82, *p* = 0.09), which did not show statistically significant effects. In the PCG studies, the SMD was 0.63 (CI: 0.33–0.93, *p* < 0.001), indicating statistical significance.

## 4. Discussion

This study was a systematic review and meta-analysis of RCT research in order to investigate the effect of ICT interventions for older adults with MCI in the local community.

In this systematic review, cognitive interventions focusing on cognitive training such as the memory, judgment, spatial perception, attention, and language skills of older adults were studied. VR devices, tablets, computers, gaming devices such as a Nintendo Wii or Xbox were used; however, the common point of all studies was that cognitive training in the form of a game was applied using ICT devices. Traditional cognitive interventions are common in group settings using cards, board games, and pen and paper, which include social interaction. However, cognitive intervention using ICT focuses more on individual cognitive training than on group interaction [48].

In the selected studies, a frequency of intervention 3 times a week or less, a session time of 60 min, and total intervention duration of 12 weeks to 6 months were the most adopted. A systematic literature review on computerized cognitive training (CCT) for normal older adult subjects showed that cognitive interventions may be ineffective in short sessions of less than 30 min, because synaptic plasticity may occur after stimulation for more than 30–60 min [49,50,51]. In addition, in the case of training more than three times per week, it was found that cognitive fatigue could impair training gains [52]. However, in the selected individual research literature, the rationales for selecting the period, frequency, or duration were not disclosed. If more RCT studies applying cognitive intervention using ICT are performed in the future, the appropriate intervention frequency could be identified through a meta-analysis study that compares the effect size of each study by frequency, session time, and period.

The outcome variables of the selected studies can be broadly classified into cognitive function, activities of daily living (ADL), emotional state (depression, anxiety, self-esteem), quality of life, motor ability, and neurophysiological images (MRI, EEG) of the brain. The above measurement variables are presented as areas that can be improved by cognitive interventions using ICT. Among the selected papers, quantitative synthesis or qualitative reasoning could not be performed because except for cognitive function, the tools for assessing ADL, depression, anxiety, quality of life, and exercise ability were different for each study. In future intervention studies in this area, for the integration of knowledge, it is necessary to apply widely used tools which have been validated for older adults in the community.

The overall purpose of this study was to determine whether cognitive intervention using ICT is an effective nursing strategy for older adults through a meta-analysis that quantifies the overall effect size. Therefore, only studies with synthetic tools and valid control group were included in the meta-analysis. As a result, a total of seven studies were analyzed. The effect size of the cognitive intervention using ICT for older adults with MCI was found to be 0.4547 (SMD) (95% CI: 0.1958–0.8500), which is small effect size according to Cohen’s criteria. In conclusion, when cognitive intervention was implemented using various devices such as iPad and VR for older adults with MCI in the community, it is encouraging that we found a small effect size despite the difference in duration and frequency of the program.

We found from the subgroup analysis that both multidomain intervention and single cognitive intervention using ICT had significant effects on cognitive function, but multidomain intervention had a larger effect size on cognitive function. Additionally, when comparing the ACG (cognitive intervention without ICT) group and the cognitive intervention group using ICT, there was no significant difference in effect size. This means that compared with traditional cognitive interventions, cognitive interventions using ICT cannot be said to have a more significant effect on older adults with MCI, according to the research accumulated so far. However, because comparing with PCG showed a significant effect, it can be said that there is a clear effect on cognitive function.

The effect size reported in a previous meta-analysis of multidomain cognitive and exercise intervention for older adults using the face-to-face method was SMD = 0.39 (*p* < 0.001), SMD = 0.39 (*p* < 0.001), SMD = 0.36 (*p* < 0.001) for older adults with dementia [9]. In another meta-analysis conducted in 2015, the SMD was 0.35 (95% CI 0.06–0.64) in the cognitive stimulation group using interaction or recall therapy compared to the active control group. However, standardized repeated cognitive practices did not report a statistically significant effect size compared to the control group [53]. A meta-analysis is a synthesis of pre-existing results; therefore, it is not appropriate to use SMD as an absolute numerical value to compare with previous meta-analysis studies. Thus, it cannot be stipulated that cognitive intervention using ICT is more effective than traditional face-to-face cognitive intervention. Notably, cognitive intervention using ICT showed a small effect size for older adults with MCI, and exhibited a statistically significant result similar to the cognitive intervention using traditional face-to-face method.

The limitation of this study is that only English and Korean literature was analyzed. There were fewer than 10 papers from which quantitative results could be synthesized. Thus, it was not possible to conclude whether the results were biased, because the publication bias could not be assessed. The accumulation of high-quality evidence in the field is necessary to make efficient estimates. In particular, because ICT is an intervention strategy, RCT research using existing traditional cognitive function training programs as a control group should be repeatedly performed before it can be applied to practice.

## 5. Conclusions

This study was conducted to investigate the effectiveness of ICT-based cognitive training program for older adults with MCI. From our review of 11 studies, it was found that cognitive intervention using ICT showed a small effect size on improvements in cognitive function and was a statistically significant result. Therefore, we conclude that cognitive training using ICT can be used in nursing practice as an effective strategy for improving and maintaining the cognitive function of older adults.

## Figures and Tables

**Table 1:** Research questions (PICOTS-SD).

Category	Research Questions
Participants	Older adults
Intervention	Information communication technology (ICT)
Comparison	-
Outcome	Cognition
Time	-
Setting	Community
Study Design	Experimental study

**Table 2:** Search terms.

AND			OR				
			**Key Term**				**Added Terms**
			**MeSH**	**EMTREE**	**CINAHL SUBHEADINGS**	**KISS, RISS**	
	Mild Cognitive Impairment (P1)		Cognitive Dysfunction [F03.615.250.700]	Cognitive defect	Cognition Disorders	Cognitive Impairment	Cognitive Impairment Mild Cognitive Impairment Mild Neurocognitive Disorder Cognitive Decline Mental Deterioration Cognition Disorder Cognitive Deficit Cognitive Disability
	Older adults (P2)		Aged [M01.060.116.100]	Aged	Aged	Elderly	Elderly Senium
	Training based on ICT (I)		Information Technology [L01.479]	Information technology	Information technology	ICT	Information and Communication Technology ICT Technology Self-Help Devices Device Telecare Electronic Game Computer Web-Based Robot
	Filter		Study with title, abstract which included above key terms Species: Human Year: 2010–2021 Study Design: RCT Language: English, Korean				

**Table 3:** Study characteristics.

Study ID	Country /City	Setting	Design /Arms	Population, N (Exp., Cont.)	Mean Age (Exp., Cont.)	Percent Female (%)	Education	Inclusion Criteria	Exclusion Criteria
(1) Anderson–Hanley, 2018	USA /New York	Comm.	RCT/3	111 (46, 45, 15)	80.9 y, 75.4 y, 78.1 y	57, 43, 50	14.9 y; 16.6 y; 15.7 y	MoCA < 26; MCI	ND; Functional limitations
(2) de Souto Barreto 2021	France /Toulouse	Comm.	RCT/2	120 (60, 60)	75.2 y, 73.2 y	31, 38	College 61.7%, 66.7%	MMSE ≥ 24; Easy access to internet; SMC	Illness; Dementia; Parkinson; Depression; Dependency ≥ 1 ADL
(3) Djabelkhir 2017	France /Paris	Comm.	RCT/2	20 (10, 10)	78.2 y, 75.2 y	60, 70	College 44.4%, 60.0%	MMSE > 24; SMC	PD and ND; Alcohol or other substance abuse; Sensory and/or motor deficits
(4) Gooding 2016	USA /New York	Comm.	RCT/3	74 (31, 23, 20)	(total) 75.59 y	(total) 32	(total)15.14 y	MMSE > 24; SMC; Normal independent functioning	-
(5) Li 2019	China/Shanghai	Comm.	RCT/2	141 (78, 63)	69.5 y, 71.5 y	33, 42	13.8 y, 13.5 y	MCI; GDR = 0.5	Stroke; Parkinson; HIV; Mood problems; Poor vision or hearing
(6) Liao 2019	Taiwan /Taipei	Comm.	RCT/2	42 (21, 21)	75.5 y, 73.1 y	61, 75	9.3 y, 9.9 y	MMSE ≥ 24; MoCA < 26; SMC; ADLs	Dementia; Malignant tumors; ND or orthopedic disease; Education < 6 years
(7) Oh 2021	Korea /Cheongju	Comm.	RCT/2	24 (12, 12)	77.8 y, 79.0 y	56, 100	4.2 y, 3.7 y	CDR = 0.5 or GDS = 2 or MCI	Hearing or visual impairment; ND or musculoskeletal disorder; Depression; Stroke; Parkinson’s
(8) Park 2018	Korea /Wonju	Comm.	RCT/2	89 (39, 39)	66.7 y, 67.6 y	63, 44	8.54 y, 8.74 y	MMSE ≥ 24; SMC; IALD ≥ 8.2	ND, PD; KGDS > 19; Auditory, visual, motor, or language impairments; Participation in CCT within 3 months
(9) Phirom 2020	Thailand /Chiang Mai	Comm.	RCT/2	40 (20, 20)	70.2 y, 69.4 y	85, 80	12.79 y, 11.20 y	Walk without a walking aid for at least 10 m; step unassisted in different directions	MMSE < 23; TGDS > 6/15 points; Health problems affecting stepping ability; Unstable health conditions
(10) Poptsi 2019	Greece	Comm.	RCT/5	100 (20, 20, 20, 20, 20)	67.9 y, 70.1 y, 71.8 y, 65.7 y, 68.1 y	64, 78, 50, 73, 71	12.14 y, 11.17 y, 9.70 y, 11.13 y, 10.36 y	aMCI; GDS stage 3; independent walking.	ND; Severe depression or anxiety; Uncontrolled hypertension or terminal illness; Cardiovascular disease; Mental illness; Pharmacological treatment; sensory deficits
(11) Thapa 2020	Korea /Busan	Comm.	RCT/2	68 (34, 34)	72.6, 72.7	82, 71	9.3 y, 4 y	MCI	PD and ND; Dementia; Dizziness; Otolaryngological disease

Exp.: Experimental group; Cont.: Control group; RCT: Randomized Controlled Trials; MoCA: Montreal Cognitive Assessment; MCI: Mild Cognitive Impairment; ND: Neurologic Disorders; comm.: community; MMSE: Mini Mental State Examination; SMC: Subjective Memory Complaints; ADL: Activities of Daily Living; PD: Psychiatric Disorders; GDR: Clinical Dementia Rating; HIV: Human Immunodeficiency Virus; CCT: Computerized Cognitive Training; GDS: Global Deterioration Scale.

**Table 4:** Contents of cognitive interventions.

Study ID	Experimental Group	Control Group	Device	Setting	Training Dose per Session (min)	Time (Hours)	Duration (Weeks)	Frequency (Weeks)	Instructor
(1)	Aerobic and Cognitive Exercise Study (ACES) Exer-score (physical exercise interactive with a relatively effortful, high cognitive demand, videogame)	Exer-tour	Recumbent stationary bike, videogame	Individual	20	-	12–16	At least 2	-
(2)	Web multidomain platform focused on three lifestyles: nutritional advice, and exercise and cognitive training	Only the wrist-worn accelerometer, information about multidomain activities	Tablet/ a wrist-worn accelerometer	Individual	-	-	24	2	-
(3)	Computerized cognitive stimulation (A web-based platform which provided several applications, i.e., appointment and event reminding, cognitive games, communication, entertainment, videos and library)	Computerized cognitive engagement	Android tablet-PC/iPad	Group (5–7 people)	90	12	12	1	Neuropsychologist
(4)	Computerized Cognitive Training (Repeated drill-and-practice exercises involving memory, attention, and executive functions within domain-specific training modules)	Cognitive vitality training, active control (game, puzzle, sudoku)	Computer	Individual or group	60		16	2	-
(5)	Multi-model computerized cognitive training at home online (Visual working memory, 30-s memory, Episodic memory, Speed of calculation, Visual search, Alertness, Mental rotation, and Images re-arrangement task)	None	Computer	Individual	40	72–96	24	3–4	-
(6)	VR-based physical and cognitive training (VR games was based on actual IADL, such as shopping, food preparation, handling finances, and transportation.)	Combined physical and cognitive training	VR (glasses on their heads and motor controllers in both hands)	Group (3–4 people)	60	36	12	3	Physiotherapists
(7)	Nurse-led computer cognitive training	Therapist-led CCT	Computer	Individual	30	12–20	4	3–5	Nurses
(8)	Using Nintendo Wii for improving functional performance	CCT	Nintendo Wii	Individual	10	30	10	3	-
(9)	Interactive physical–cognitive game - Physical part: (1) Stepping on different targets and in different directions (2) Balancing - Cognitive part: (1) Executive function (2) Attention, (3) Memory	Educational material	Xbox 360 Kinect Sensor V2, LED projector, computer	Individual	60	36	12	3	Researchers
(10)	(1) Paper and pencil group (PP/G) (2) Computer-based program of language tasks group (PC/G) (3) Oral group (OR/G) (Semantic expression of language, semantic comprehension of language and phonemic expression of language)	(4) Active control group: Unstructured session with discussion of current events (5) Control group: None	Computer	Group (5–10 people)	60	48	24	2	Expert Psychologists
(11)	VR-based cognitive training (Juice making, Crow shooting, Find the fireworks number, Memory object at the house) + Educational program	Educational program	Oculus VR headset, two wireless hand controllers	Individual	100	24	8	3	-

**Table 5:** Outcome measurements and results.

Study ID	Outcome Measurements and Results							
	Global Cognitive	Attention	Memory	Verbal Fluency	Executive Function	Visuospatial Ability	Motor Skill	Etc.
	Tool(*p*)							
(1)	MoCA(0.79)	Digit span (0.50)	Verbal Memory(0.047^delay^)	-	Stroop test (0.001), Color trails (0.32)	-	Get up and go (0.46)	MRI, Saliva, Protein
(2)	MMSE(0.71) WAIS-R(0.09)	COWAT (0.15)	FCSRT (0.13) CNT (0.12)	-	-	-	SPPB (0.30); Gait speed (0.11)	GDS (0.21), MNA (0.61), HRQOL (0.04)
(3)	MMSE(0.35)	Digit span (0.74)	-	Verbal fluency (0.4)	TMT-(0.23^A^,0.29^B^)	VST (0.46)	-	Anxiety (0.49), Depression (0.64), Self-esteem (0.76), CDS (0.80)
(4)	MMSE(<0.001)	-	BSRT (<0.01) VR (0.52^I^,0.09^II^) LM-Ⅱ(0.01)	-	-		-	BDI (0.04)
(5)	MMSE(<0.05) ACER (<0.05^memory,fluency,language^)	-	AVLT immediate, (<0.1^20 min^ ^recall^)	-	Stroop (<0.05^index^), STT (n.s.), SDS (n.s.)	CFT recall (n.s.) CFT copy (<0.05)	-	MRI
(6)	MoCA(0.181)		CVVLT immediate (0.149^immediate^,0.115^delay^)	-	EXIT-25(0.724)	-	-	EEG, IADL (0.006)
(7)	MMSE(0.999)	Digit Span (0.594^forward^,0.729^backward^)	SVLT-E (0.030^immediate^) K-BNT (0.012)	-	K-CWST (0.375^word^,0.205^color^)	RCFT (0.231)	-	EEG; IADL (0.352)
(8)	-	Digit Span (< 0.05)	RAVLT (n.s.)	-	TMT (n.s.), Stroop Color-Word Test (n.s.)	CFT (n.s.) WAIS-BDT (n.s.)	-	HRQOL (< 0.05)
(9)	MoCA(0.001)	-	-	-	-	-	PPA (0.002), TUG (0.015^single^/0.025^dual^)	-
(10)	MMSE(n.s.)	RAVLT 1 (n.s.)	RAVLT 2 (n.s.) PPT (n.s.) RBMT (n.s.)	FAS total (n.s.) BDAE (n.s.)	-	-	-	FRSSD (n.s.)
(11)	MMSE(n.s.)	SDST (0.03)	-	-	TMT A(n.s.) TMT B(0.01)	-	Grip strength (n.s.), speed (0.02), 8 feet up and go (0.03)	EEG

Notes: n.s. = not significant; VS: Visuospatial Memory; MoCA: Montreal Cognitive Assessment; imm.: immediately; MRI: Magnetic Resonance Imaging; MMSE : Mini-Mental Status Examination; COWAT: Controlled Oral Word Association Test; WAIS-R: Wechsler Adult Intelligence Scale-Revised; FCSRT: Free and Cued Selective Reminding Test; CNT: Category Naming Test; SPPB: Short Physical Performance Battery; GDS: Geriatric Depression Scale; MNA: Mini Nutritional Assessment; HRQOL: Health-Related Quality of Life; TMT: Trail Making Test; CDS: Cognitive Difficulties Scale; BSRT: Buschke Selective Reminding Test; VR: Wechsler Memory Scale-Revised Visual Reproductions Subtests; BDI: Beck Depression Inventory; STT: Shape Trail Test; SDS: Symbol Digit Substitution Test; ACER: Addenbrooke’s Cognitive Examination—Revised; AVLT; Auditory Verbal Learning Test; CFT: Complex Figure Test; EXIT: Executive Interview; CVVLT: The Chinese version of the Verbal Learning Test; EEG: Electroencephalography; IADL: Instrumental Activities of Daily Living scale; RAVLT: Rey Auditory Verbal Learning Test; BDY: Block Design Test; PPA: Physiological Profile Assessment; TUG: Timed Up and Go test; FRSSD: Functional Rating Scale for Symptoms of Dementia; RBMT: Rivermead Behavioral Memory Test; FAS: Verbal Fluency test; BDAE: Boston Diagnostic Aphasia Examination; PPT: Pyramids and Palm Trees test, SDST: Symbol Digit Substitution Test.

## Data Availability

The data presented in this study are available in selected articles in the reference list.

## Supplementary Materials

The following are available online at https://www.mdpi.com/article/10.3390/ijerph182111535/s1, Table S1: Search results.
